# Diagnostic Value of Inflammatory Markers in Inflammatory Bowel Disease: Clinical and Endoscopic Correlations

**DOI:** 10.7759/cureus.84073

**Published:** 2025-05-14

**Authors:** Nazdar Omer, Shereen Ibrahim, Ali A Ramadhan

**Affiliations:** 1 Department of Medical Physiology and Pharmacology, University of Duhok, Duhok, IRQ; 2 Department of Medicine, University of Duhok, Duhok, IRQ

**Keywords:** biomarkers, crohn’s disease (cd), endoscopy, inflammatory bowel disease, inflammatory mediators, ulcerative colitis (uc)

## Abstract

Background

The incidence of inflammatory bowel diseases (IBD) has increased over the last century. Despite the unclear etiology of IBD, significant advancements have been made in recent years to understand the development of this disease.

Objectives

The aim of this study was to examine the diagnostic significance of several inflammatory biomarkers and their associations with clinical and endoscopic disease activity in patients with IBD.

Methods

This case-control study involved 68 patients, 34 with Crohn’s disease (CD) and 34 with ulcerative colitis (UC), with IBD confirmed by endoscopy and histopathology, and 52 healthy subjects as a control group. A pre-designed questionnaire was completed for each subject. Stool samples were taken for fecal calprotectin (FC) measurement by enzyme-linked immunosorbent assay (ELISA), and a blood sample was obtained for complete blood and differential counts using a hematology autoanalyzer, erythrocyte sedimentation rate (ESR), serum C-reactive protein (CRP) by Cobas (Roche Diagnostics, Basel, Switzerland), and measurements of interleukins (IL-6, IL-17A, IL-8, and IL-1β) by ELISA. Comparisons of parameters were considered statistically significant at the P < 0.05 interval.

Results

The mean levels of ESR, CRP, FC, all measured ILs, neutrophil number and percentage (%), neutrophil-lymphocyte ratio (NLR), and platelet number were statistically significantly higher in patients with IBD compared to the control group. However, lymphocyte%, lymphocyte-mid-size leukocytes ratio (LMR), hemoglobin, mean corpuscular hemoglobin (MCH), hematocrit, and red cell distribution width (RDW) were significantly lower in patients with IBD compared to controls. Receiver operating characteristic (ROC) curve analyses showed that FC, IL-8, and ESR, respectively, have the highest area under the curve (AUC) and were of the highest validity to differentiate between patients with IBD and healthy subjects, followed by CRP and IL-1β.

Conclusion

All the studied ILs were increased in patients with IBD; their increases were not different between UC and CD patients. FC, IL-8, and ESR were of the highest validity for the diagnosis of IBD. Findings collectively highlight the validity of the measured parameters as potential biomarkers in IBD.

## Introduction

Inflammatory bowel disease (IBD), encompassing ulcerative colitis (UC) and Crohn's disease (CD), is a chronic and recurring disorder affecting the gastrointestinal (GI) tract. It is characterized by abdominal pain, chronic diarrhea, and blood in the stool. In North America and Europe, approximately 3.7 million individuals are affected by IBD. Moreover, there has been a notable rise in the number of cases of IBD in Asian countries, including China, India, South Korea, and Saudi Arabia, during the last two decades. During the second part of the 20^th^ century, IBD became much more common, and since the turn of the millennium, it has been ranked among the most common GI disorders. Therefore, the costs of care for IBD have increased in recent years and are driven by specific therapeutics and disease features [1].

Despite the unclear etiology of IBD, significant advancements have been made in recent periods to understand the pathophysiology of this disease. Research has demonstrated that the development of IBD is linked to the genetic susceptibility of the individual, the composition of the intestinal microbiota, several environmental factors, and abnormalities in the immune system. Individuals with a positive family history of IBD are more prone to having the condition. However, the severity of CD is not influenced by this history. In Western countries, 10% to 25% of IBD cases have a favorable family history. In Asia, however, this percentage is substantially lower, at less than 5%. A study conducted on a group of German twins found that the concordance rates in the presence of IBD were 35% for CD and 16% for UC, indicating that genetics plays a significant role in the development of these conditions. Research has discovered more than 240 distinct gene alleles involved in the pathogenesis of the disease, with approximately 30 being common to both types of IBD [2]. Moreover, the frequency of genetic variations linked to IBD can shift depending on the ethnic composition; it fluctuates among various racial and ethnic groups.

A variety of environmental factors, such as stress, dietary habits, and antibiotic use, contribute to the development of IBD. These factors lead to an imbalance in the gut microbiota, known as dysbiosis. Dysbiosis then affects the integrity of the gut barrier, as well as the innate and adaptive immune responses. This results in uncontrolled chronic inflammation and the over-activation of T helper 1 (Th1) and 17 (Th17) cells. Additionally, dysbiosis leads to an increase in the permeability of tight junctions, a reduction in regulatory T (Treg) cells, and a decrease in the production of regenerating islet-derived 3 gamma (REG3γ) and IL-10, which are important molecules involved in immune regulation [3].

Several proinflammatory cytokines and chemokines are currently recognized as significant contributors to the development of IBD. The immunological components of IBD encompass compromised innate and adaptive immunity, which can be linked to genetic vulnerability, environmental factors, and the bacteria in the intestines. Th17 cells have a significant effect on both the development and the persistence of the disease. Furthermore, malfunctioning anti-inflammatory mechanisms, such as a reduction in Treg cells, also have a role in sustaining the condition. Furthermore, our understanding of the distinct roles of immune cells in this entire process has been altered due to recent findings [4].

IL-6 was initially discovered as a substance produced by Th2 cells almost four decades ago. IL-6 is crucial in the development of IBD. Its biological action is exerted through signaling either through adhering to its receptors or by trans-signaling, which occurs when it binds to a soluble form of the IL-6 receptor and then to the membrane-bound glycoprotein 130 [5].

In recent studies, the investigation of the development of IBD has primarily centered on Th17 cells, which are responsible for producing IL-17. Initially, IL-17 was believed to be generated only by T cells; however, it is currently understood that it is also released by many innate cells. The IL-1 family of cytokines has a fundamental effect on the development of IBD. Investigations have observed increased IL-1β formation by monocytes in the colon lamina propria of patients with active IBD. Furthermore, there is a correlation between IL-1β levels in the colon and disease activity, with increased IL-1β correlated with active lesions [6].

Mounting evidence suggests that CXC-chemokine ligand 8 (CXCL8) participates in leukocyte transmigration into tissues, including adhesion, migration, and activation of neutrophils. The upregulation of CXCL8 expression in the colonic mucosa of individuals with active UC compared to healthy individuals has been extensively studied, and a significant association has been found between CXCL8 levels and the severity of inflammation. However, the available data on CXCL8 expression in CD are limited and conflicting [7].

The discovery of fecal calprotectin (FC) dates back to 1980. Several GI disorders, including IBD, can cause an increase in FC concentrations. Various studies support the utilization of FC in IBD diagnosis. The FC test shows promise as a regular screening method and could be a valuable tool in distinguishing between Crohn's disease and irritable bowel syndrome (IBS). Due to the less-than-ideal performance of existing biomarkers, endoscopy with biopsies is still considered the most reliable method for assessing and tracking inflammatory activity. However, its usage is limited because it is invasive and requires collecting specimens as needed. Therefore, it is imperative to urgently seek widely available and cost-efficient biomarkers for evaluating the disease activity [8].

An optimal laboratory biomarker should possess ease of measurement, rapidity, reproducibility, specificity, and prognostic value. An optimal marker for IBD would significantly simplify the tasks of gastroenterologists and surgeons who treat these patients. Over the past few decades, significant research has focused on identifying non-invasive and dependable biomarkers of disease severity in IBD. These biomarkers should be easily detectable in bodily fluids without causing any negative effects on the patient's quality of life. Regrettably, the search for the perfect biomarker is still ongoing, and a new investigation explored the importance of enhancing measurement accuracy by integrating multiple indicators [9].

The present study aimed to assess and validate a comprehensive panel of inflammatory biomarkers to improve the non-invasive diagnosis of IBD in a cohort of patients with IBD and healthy subjects. Furthermore, to examine the associations between these inflammatory biomarkers and the level of disease activity in patients with IBD, evaluated through clinical assessments and endoscopic procedures.

## Materials and methods

Study design and subjects

This case-control study was carried out at the Department of Medical Physiology and Pharmacology, College of Medicine, University of Duhok, and at the Duhok GI and Hepatology Center and Jeen Private GI Clinic, all located in Duhok, Kurdistan Region, Iraq. The approval was provided by the Research Ethics Committee of the College of Medicine, University of Duhok (approval number: 18082021-8-13). Formal consent was obtained from each participant at the start of the sample collection phase.

Sixty-eight patients with endoscopically confirmed IBD were recruited for the study. This group included 39 males and 29 females with a mean age of 30.51 ± 13.21 years; among them were 34 patients with CD and 34 with UC. The control group consisted of 52 healthy subjects recruited randomly from the general population. The subjects included 30 males and 22 females with a mean age of 34.37 ± 13.24 years.

All patients in all age groups with newly diagnosed and endoscopically-approved IBD were included in this study. However, subjects with pregnancy, breastfeeding, current inflammatory or autoimmune disease, other GI tract diseases, and a history of antibiotic therapy in the last three months were excluded from the study.

Data collection and classification of patients

All participants (after their approval) completed a pre-tested questionnaire that included history, anthropometric data, and clinical examination. The IBD group was subdivided into patients with CD and with UC.

Body mass index (BMI) measurement

The height and weight of each individual were measured to calculate BMI using the following formula: BMI (kg/m²) = weight (kg) / height (m²). Individuals with a BMI below 18.5 kg/m² were classified as underweight, those with a BMI between 18.5 and 24.9 kg/m² were deemed normal, those with a BMI from 25 to 29.9 kg/m² were considered overweight, and individuals with a BMI of 30 kg/m² or higher were categorized as obese.

Study protocol

Following the confirmation of the IBD diagnosis and the completion of a pre-tested questionnaire, a 10 ml venous blood sample was collected from an appropriate antecubital vein. Of this, 3 ml was transferred into an ethylenediamine tetraacetic acid (EDTA)-containing tube for the measurement of complete blood count (CBC) and erythrocyte sedimentation rate (ESR). The remaining 7 ml of blood was placed in a plain plastic tube and then centrifuged at 3000 rpm for 20 minutes. The obtained serum was stored at -20°C for the determination of inflammatory biomarkers. A fresh stool sample was obtained from each patient for immediate measurement of FC.

Evaluation of disease activity and severity by endoscopy

After proper preparation of the patients for endoscopy, this procedure was performed using Olympus EVIS EXERA III (Olympus Medical Systems, Tokyo, Japan). Participants diagnosed with IBD through endoscopic evidence were included in the study. We assessed disease activity and severity using the Crohn's Disease Activity Index for CD [10] and the Mayo score for UC severity [11].

Measurement of ESR

The principle of ESR is when an anticoagulant is added to the blood and mixed, then placed in a vertical tube; red blood cells (RBC) tend to settle toward the bottom of the tube, providing clear plasma on top. This rate of RBC sedimentation in a given interval of time is called ESR. The Westergren method was used for ESR estimation. For this purpose, 1.6 ml of blood was mixed with 0.4 ml of sodium citrate (ratio 4:1) and mixed well, then drawn into a Westergren tube to the 200 mm mark. The tube was placed in a rack in a vertical position for one hour at room temperature. At the end of this time, the distance from the lowest point of the surface meniscus to the upper limit of RBC sediment was measured and expressed as mm/1 hour.

Measurement of inflammatory mediators

A panel of inflammatory cytokines was used as potential biomarkers for IBD, including interleukin-1β (IL-1β), IL-17, and CXCL8. The serum concentrations of the aforementioned cytokines were measured by enzyme-linked immunosorbent assay (ELISA) using commercially available kits (SunLong Biotech Co., Ltd., Hangzhou, China). IL-6 was measured by using a chemiluminescence immunoassay (CLIA) kit, which is a specialized kit for the quantitative determination of IL-6 using the MAGLUMI series fully automated CLIA analyzer (Snibe Ltd., Shenzhen, China). For the assessment of serum values of C-reactive protein (CRP), Cobas (Roche Diagnostics, Basel, Switzerland) was used to quantitatively measure CRP in human serum and plasma in vitro.

Measurement of FC levels

FC levels were measured using the Alegria® calprotectin (Orgentec Diagnostika GmbH, Mainz, Germany), an automated, in vitro test system for the quantitative determination of calprotectin in stool. The product is intended for professional in vitro diagnostic use.

Statistical analysis

The statistical analyses were performed using IBM SPSS Statistics software, version 26 (IBM Corp., Armonk, NY). The statistical significance of the mean difference was assessed by an independent samples t-test. A chi-square test was employed to compare differences expressed in percentages. Pearson's linear correlation coefficient was used to evaluate the significance, power, and trend of proportional association between two strictly evenly distributed parameters. Utilizing receiver operating characteristic (ROC) curve analysis to calculate area under the curve (AUC) for the evaluated parameters in the given setting of comparing the groups and diagnostic cut-off value for each parameter, the ROC area ranges from 0.5 to 1. P-values of less than 0.05 were considered significant.

## Results

Table 1 shows the demographic features of the study participants. Subjects were categorized into the study group (IBD, n=68) and apparently healthy controls (n=52). Findings indicated that no statistically significant differences in the means and/or percentage of compared demographic parameters between apparently healthy controls and the IBD group were detected. 

**Table 1:** Demographic parameters of the study groups Results are expressed as mean ± standard deviation. The independent t-test was conducted to compare the means between two distinct groups. The p-value was calculated to assess the statistical significance of the differences observed between these groups. A p-value of ≤0.05 was considered statistically significant, while a p-value of ≤0.01 was regarded as highly statistically significant. IBD: inflammatory bowel disease

Parameters	Groups: Healthy controls (N=52) and IBD (N=68)	Mean ± SD	P-value
Age	Healthy controls	34.37 ± 13.24	0.994
	IBD group	30.51 ± 13.21	
Weight (Kg)	Healthy controls	68.83 ± 14.22	0.876
	IBD group	62.92 ± 14.11	
Height (m)	Healthy controls	1.64 ± 0.09	0.246
	IBD group	1.65 ± 0.12	
BMI (Kg/m^2^)	Healthy controls	25.23 ± 4.3	0.938
	IBD group	22.86 ± 4.16	
Gender (Male/Female)	Healthy controls	30 / 22 (57.7/43.3%)	0.447
	IBD group	39 / 29 (57.3/42.7%)	

Table 2 illustrates the demographic features of the IBD group. As previously described, the study group was subdivided into CD and UC. The mean age and weight of patients with UC were significantly higher than those of patients with CD (P = 0.02 and 0.009). However, there were no significant differences in the means of BMI and height between the two groups.

**Table 2:** Demographic parameter of patient subgroups Results are expressed as mean ± standard deviation. The independent t-test was conducted to compare the means between two distinct groups. The p-value was calculated to assess the statistical significance of the differences observed between these groups. A p-value of ≤0.05 was considered statistically significant, while a p-value of ≤0.01 was regarded as highly statistically significant.

Parameters	Mean ± SD Crohn’s disease (N=34)	Mean ± SD Ulcerative colitis (N=34)	P-value
Age (years)	28.18 ± 10.68	32.85 ± 15.24	0.02
Weight (kg)	60.27 ± 10.97	65.49 ± 16.36	0.009
Height (cm)	1.67 ± 0.089	1.63 ± 0.14	0.145
Body mass index (kg / m^2^)	21.55 ± 3.74	24.13 ± 4.2	0.418

A panel of inflammatory and hematological parameters was measured as potential biomarkers for IBD (Table 3). Statistically significant elevation in ESR, CRP, FC, IL-8, IL-1β, IL-17A, IL-6, white blood cell (WBC) count, mid-size leucocyte number, neutrophil%, neutrophil number, neutrophil-lymphocyte ratio (NLR), and platelet number were observed in patients with IBD compared to controls. Moreover, there were significantly lower mean levels observed for lymphocyte count and percentage, lymphocyte-to-monocyte ratio (LMR), hemoglobin, mean corpuscular hemoglobin (MCH), mean corpuscular volume (MCV), hematocrit, and red cell distribution width (RDW). However, the difference in the mean percentage of mid-size leukocytes did not achieve statistical significance.

**Table 3:** Comparison of selected parameters between the control group and IBD group Results are expressed as mean ± standard deviation. The independent t-test was conducted to compare the means between two distinct groups. The p-value was calculated to assess the statistical significance of the differences observed between these groups. A p-value of ≤0.05 was considered statistically significant, while a p-value of ≤0.01 was regarded as highly statistically significant. IBD: inflammatory bowel disease

Parameters	Healthy control group (N=52) Mean±SD	IBD group (N=68) Mean± SD	P-value
Erythrocyte sedimentation rate (mm/1hr)	10.54±6.921	37.40±23.98	0.000
C–reactive protein (mg/l)	2.41±2.12	32.62±40.1	0.000
Fecal calprotectin (μg/g)	22.61±18.81	592.17±303.3	0.000
Interleukin–8 (pg/ml)	82.31±35.023	189.34±120.21	0.000
Interleukin-1β (pg/ml)	43.9±35.78	94.29±59.37	0.000
Interkeukin-17A (pg/ml)	47.97±32.1	78.4289±62.01	0.002
Interleukin-6 (pg/ml)	3.85±2.025	12.2±19.59	0.003
White blood cell count (10^3^ / mm^3^)	7.42±1.76	8.31±2.87	0.052
Lymphocyte number (10*9/L)	2.54±0.76	2.25±1.04	0.098
Lymphocyte percentage (%)	34.62±6.50	27.38±10.17	0.000
Mid-size leukocyte number (10*9/L)	0.415±0.26	0.524±0.4	0.096
Mid-size leukocyte percentage (%)	6.20±2.69	5.53±3.20	0.225
Neutrophil number (10*9/L)	4.410±1.2	5.860±2.37	0.000
Neutrophil percentage (%)	59.165±7.25	67.19±11.11	0.000
Neutrophil-lymphocyte ratio	1.87±0.64	3.18±2.44	0.000
Lymphocyte-mid-size leukocytes ratio	7.52±4.05	5.64±2.69	0.003
Hemoglobin level (gm/dl)	13.86±1.71	12.15±1.96	0.000
Mean corpuscular hemoglobin (pg)	27.72±2	26.04±3.4	0.002
Mean red blood cell volume (fl)	84.431±5.58	81.71±8.61	0.050
Hematocrit (%)	42.26±5.04	38.99±5.23	0.001
Red blood cell distribution width (fl)	51.55±10.71	37.01±18.87	0.000
Platelet number (10*9/L)	220.52±50.71	314.16±118.36	0.000

To further explore the validity of the measured biomarkers, the IBD group was subdivided into CD and UC. Patients with CD showed significantly higher mean levels of ESR, CRP, and platelet count compared to their mean levels in patients with UC (Table 4). Comparisons of all other selected parameters between the two subgroups of patients turned out to be non-significant.

**Table 4:** Comparison of selected parameters between patients with Crohn’s disease and ulcerative colitis Results are expressed as mean ± standard deviation. The independent t-test was conducted to compare the means between two distinct groups. The p-value was calculated to assess the statistical significance of the differences observed between these groups. A p-value of ≤0.05 was considered statistically significant, while a p-value of ≤0.01 was regarded as highly statistically significant.

Parameters	Mean ± SD Crohn’s disease (N=34)	Mean ± SD Ulcerative colitis (N=34)	P-value
Erythrocyte sedimentation rate (mm/1hr)	47.33 ± 25.18	27.76 ± 18.48	0.001
C–reactive protein (mg/l)	48.1 ± 47.04	17.59 ± 24.44	0.001
Fecal calprotectin (μg/g)	573.11 ± 296.93	610.66 ± 312.66	0.616
Interleukin–8 (pg/ml)	201.17 ± 137.6	178.86 ± 109.53	0.465
Interleukin-1β (pg/ml)	96.44 ± 50.33	94.8 ± 68.72	0.912
Interkeukin-17A (pg/ml)	84.63 ± 75.52	74.28 ± 50.38	0.511
Interleukin-6 (pg/ml)	15.22 ± 25.29	9.26 ± 11.36	0.215
White blood cell count (10^3^ / mm^3^)	8.73 ± 2.68	7.9 ± 3.03	0.242
Lymphocyte number (10*9/L)	2.21 ± 0.98	2.29 ± 1.1	0.753
Lymphocyte percentage (%)	26.07 ± 10.27	28.66 ± 10.06	0.302
Mid-size leukocyte number (10*9/L)	0.5 ± 0.46	0.54 ± 0.33	0.712
Mid-size leukocyte percentage (%)	5.17 ± 3.45	5.87 ± 2.94	0.373
Neutrophil number (10*9/L)	6.31 ± 2.41	5.41 ± 2.27	0.120
Neutrophil percentage (%)	69.11 ± 11.83	65.32 ± 10.2	0.165
Neutrophil-Lymphocyte ratio	3.71 ± 3.16	2.67 ± 1.3	0.082
Lymphocyte-mid-size leukocytes ratio	5.72 ± 2.36	5.56 ± 3.02	0.815
Hemoglobin level (gm/dl)	11.9 ± 1.91	12.39 ± 2.01	0.311
Mean corpuscular hemoglobin (pg)	26.11 ± 3.5	25.96 ± 3.36	0.858
Mean red blood cell volume (fl)	81.66 ± 8.51	81.75 ± 8.83	0.967
Hematocrit (%)	38.16 ± 5.73	39.79 ± 4.64	0.203
Red blood cell distribution width (fl)	39.26 ± 18.04	34.81 ± 19.66	0.339
Platelet number (10*9/L)	347.12 ± 124.89	282.18 ± 103.69	0.024

The ROC analysis between selected variables was performed to differentiate between apparently healthy controls and IBD group (Table 5). FC showed the highest AUC (0.990, P <0.0001) with a cut-off value > 75.1 μg/g, sensitivity of 97.1 %, and 100 % specificity to differentiate between IBD and healthy subjects. The following parameter with a higher AUC was IL-8; cut-off value > 111 pg/ml, sensitivity of 95.77 % and 90.39%. AUC, sensitivity, and specificity for both ESR and CRP were closer to each other with cut-off values of > 25 mm/1 hour and > 6.3 mg/l, respectively (P <0.0001). The AUC for IL-1β was 0.806, and the cut-off value was > 47.2 pg/ml, with higher sensitivity (87.32 %) but lower specificity (61.54 %) than ESR and CRP. The other parameters listed in Table 5 were also essential in distinguishing between individuals with IBD and healthy subjects. Nevertheless, they exhibited lower AUC values and/or reduced sensitivity and specificity.

**Table 5:** ROC analysis of selected parameters between IBD and control subjects Results are expressed as AUC, cut-off, specificity %, and sensitivity %. A p-value of ≤0.05 was considered statistically significant, while a p-value of ≤0.01 was regarded as highly statistically significant. AUC: area under the curve; ROC: receiver operating characteristic; IBD: inflammatory bowel disease

Parameters	AUC	Cut-off value	Sensitivity %	Specificity %	P –value
Erythrocyte sedimentation rate (mm/1hr)	0.867	> 25	62.69	100	< 0.0001
C–reactive protein (mg/l)	0.838	> 6.3	67.16	98.08	< 0.0001
Fecal calprotectin (μg/g)	0.990	> 75.1	97.01	100	< 0.0001
Hematocrit (%)	0.671	≤ 38.8	53.73	75	< 0.0014
Hemoglobin level (gm/dl)	0.737	≤ 12	47.76	86.54	< 0.0001
Interkeukin-17A (pg/ml)	0.754	> 53.9	77.46	61.54	< 0.0001
Interleukin -1β (pg/ml)	0.806	> 47.2	87.32	61.54	< 0.0001
Interleukin-6 (pg/ml)	0.716	> 7.56	53.73	96.15	< 0.0001
Interleukin – 8 (pg/ml)	0.949	> 111	95.77	90.38	< 0.0001
Lymphocyte-mid-size leukocyte ratio	0.670	≤ 7.2	80.60	50	0.0015
White blood cell count (10^3^ / mm^3^)	0.585	> 7.1	68.66	51.92	0.1134
Red blood cell distribution width (fl)	0.702	≤ 34.6	52.24	98.08	0.0002
Neutrophil-lymphocyte ratio	0.733	> 2.11	67.16	76.92	< 0.0001

## Discussion

IBD is a global health issue, as evidenced by the consistent growth in the publication of IBD-related research throughout the last 10 years. The diagnostic efficacy and limitations of endoscopy and inflammatory biomarkers in IBD have been thoroughly investigated over the years, leading to an enhanced understanding of their significance in clinical settings. However, the integration of endoscopy and molecular tests has become a robust diagnostic method for IBD, and ongoing efforts are in progress to identify an optimal diagnostic tool that may address the limitations of the existing methods. Lately, there has been an increasing interest in transitioning from employing a single biomarker to the biomarker panel method to identify biomarkers that are collectively unique to inflammatory IBD and can facilitate the distinction between UC and celiac disease [12].

Concerning the demographic features of the recruited subjects, there is apparent compatibility in age, BMI, and gender between the two study groups (healthy controls and IBD group), indicating that the obtained results are not related to the impact of these factors. During the study period, 68 patients were newly diagnosed with IBD; the number of patients with CD was 34 (50%), and was equal to that of UC (34, 50%). In contrast to our findings, A retrospective analysis conducted by Shamkh et al. identified a total of 592 newly diagnosed individuals with IBD, with 153 (26%) having UC and 439 (74%) having CD. A retrospective analysis of patients with IBD who had been diagnosed for a period of 10 years. Out of the 169 patients with IBD, 136 were confirmed patients with UC (80.5%), while the remaining 33 patients (19.5%) were diagnosed with CD [13].

In this study, patients with IBD had significantly higher mean values of the measured inflammatory biomarkers than controls. However, significantly lower mean levels of lymphocyte number and %, LMR, hemoglobin, MCH, MCV, hematocrit, and RDW were detected in patients with IBD compared to controls. An elevated WBC count is frequent in people with active IBD and does not always indicate infection. Thayer et al. attributed the elevated total WBC count in patients with IBD to increased polymorphonuclear cells in percentage and absolute number. Despite a significant reduction in lymphocytes in patients with IBD compared to the control group, there was no variation in the absolute lymphocyte count. The reduced lymphocyte percentage in IBD is due to the increased predominance of polymorphonuclear leukocytes in this condition [14].

CRP is the primary serum biomarker to assess inflammation in individuals with IBD. It has been shown that ESR, CRP, RDW, and NLR levels in patients with active IBD were significantly higher than those in patients with non-active IBD. Some researchers reported that in patients with IBD, CRP was insignificantly higher than in healthy controls, although higher levels in CD than UC were observed (7.3 ± 2.1 in controls, 16.7 ± 5.5 in CD, and 10.1 ± 7.9 in UC). Moreover, CRP did not change significantly after treatment in CD and UC. A recent study by Xu et al. in 2024 revealed that inflammatory bowel disease patients exhibit elevated platelet number and platelet count and significantly lower mean platelet volume [15].

FC is a calcium-binding protein primarily derived from neutrophils, with contributions from monocytes and macrophages. It serves as a non-invasive biomarker for gastrointestinal inflammation, particularly in IBD, including CD and UC. Elevated FC levels can also be observed in infectious colitis, diverticulitis, and colorectal cancer. The FC levels were significantly elevated in patients with IBD compared to healthy controls. Our findings are consistent with the expected high levels of FC in patients with active IBD, which can be due to the high presence of neutrophils in the GI tract, a characteristic feature of the disease [12]. The clinical recommendations propose the utilization of FC measurement as a component of the diagnostic evaluation for CD and UC. The accumulation of elevated levels of FC was observed throughout the progression of IBD. FC is valuable in identifying the underlying cause of GI symptoms, particularly in cases where distinguishing between organic and functional factors through symptoms or clinical examination proves challenging. In practical application, it is employed to differentiate between IBD and IBS despite the similarity in signs and symptoms. FC concentrations tend to be higher in the distal bowel compared to the proximal bowel due to localized inflammation. Specific FC thresholds help differentiate conditions: levels above 250 µg/g strongly indicate IBD, while lower values may suggest tuberculosis (TB) or other infectious causes. Approximately 99% of patients with active IBD exhibit increased levels of FC [16].

The innate and adaptive immune systems play a crucial role in regulating intestinal inflammation in individuals with IBD. The progression, reappearance, and worsening of the inflammatory process in IBD are interdependent on the levels of cytokines. In the development of IBD, the innate immune system is of vital importance. Upon activation, macrophages and dendritic cells (DCs) have the potential to release various cytokines that control the inflammatory process in IBD. The present investigation found that all the measured ILs were elevated in patients with IBD compared to healthy controls. Previous clinical investigations on IBD have shown significant changes in the synthesis of specific cytokines or the sensitivity of immune cells to different cytokines. Furthermore, several proinflammatory cytokines and chemokines are currently recognized as significant contributors to the development of IBD [17].

Consistent with our findings, Fauny et al (2020) found significantly higher serum levels of IL-6 and IL-17A in patients with CD, with significantly higher serum levels of IL-8 and IL-17A in patients with UC when compared with healthy controls. Several studies have shown significant changes in the levels of proinflammatory cytokines in the serum and/or GI mucosa of patients with IBD. Increased levels of IL-17, Th17 cells, and Th17-related cytokines were found in the GI mucosa of patients with IBD [18].

Studies have consistently shown that the expression of CXCL8 is higher in the colonic mucosa of individuals with active UC compared to normal control subjects. Furthermore, the level of inflammation is directly proportional to the increased concentration of CXCL8. Nevertheless, the evidence provided for CD is subject to controversy [7].

A significant portion of research on IL-1 in the context of IBD has been on IL-1β, which is elevated in IBD. The principal source of these elevated amounts is mononuclear cells, most notably macrophages of monocytic origin, which lie within the lamina propria. IL-6 is crucial in the pathogenesis of IBD. During inflammation, hepatocytes significantly enhance the synthesis of CRP under the signal of IL-6 [4]. In addition, there was a significant reduction of IL-6 levels in biologics-treated patients with IBD, which can help in predicting the response to treatment and tailoring treatment strategies [19].

Patients with CD showed significantly higher mean levels of ESR, CRP, and platelet count compared to their mean levels in patients with ulcerative colitis. Comparisons of all other selected parameters between the two subgroups of the patients turned out to be nonsignificant. Some of these findings are comparable, and others are contradictory to previous studies; the concentration of CRP in the UC group was markedly higher than in the patients with CD, while there was no significant difference in mean ESR level between UC and CD patients [20].

The researchers reported that mucosal levels of CXCL8 are considerably elevated in active UC but not in celiac disease. They hypothesized that CXCL8 may have a more substantial role in promoting inflammation in UC. Furthermore, it was reported that both active UC and CD patients exhibited elevated levels of CXCL8. Concerning IL-17A, the level of IL-17A cytokine showed no significant difference between UC and CD patients [21]. In patients with IBD, we didn’t notice a significant difference in mean FC levels between patients with UC and CD. Contrary to our results, higher levels of FC were reported in CD than in UC [22].

FC showed the highest AUC (0.990, P < 0.0001) with a cut-off value > 75.1 μg/g, sensitivity of 97.1 %, and 100 % specificity to differentiate between IBD and healthy subjects. Challenges in comparing findings from published data on the diagnostic efficacy of FC stem from varying thresholds for determining a positive test. Most studies have employed a threshold of 50 μg/g to determine a positive test outcome and to determine whether patients with stomach pain should undergo endoscopy to exclude IBD or other organic diseases. Based on calculations, using FC as a diagnostic test for suspected IBD would lead to a 67% decrease in the number of patients who require endoscopy [23].

Furthermore, to differentiate between IBS and IBD, a cutoff of 50 μg/g provided a sensitivity of 88% and specificity of 78%. Nevertheless, by applying a threshold of 100 μg/g, the sensitivity rose to 97%, while the specificity slightly decreased to 76%. Conversely, the positive predictive value (PPV) reached 75%, and the negative predictive value (NPV) reached 97% [24]. In contrast, when we used a cut-off value of > 75.1 μg/g, the sensitivity was similar to the cut-off value of 100 μg/g, and the specificity was 100%.

An established and extensively researched threshold for the existence of active inflammation in both UC and CD is 250 mg/g. Previous studies have demonstrated that FC levels above 145 mg/g accurately predict severe illness on magnetic resonance imaging (MRI) with a sensitivity of 69.3% and specificity of 71.4% [25].

Interstitial inflammation in individuals with IBD was found to be more accurately and effectively detected using FC as a biomarker compared to CRP or ESR. It may aid in the identification of patients with UC and CD who are highly susceptible to clinical relapse. In this study, in accordance with the findings of Chang et al., we also reported higher AUC for FC than CRP and ESR [26].

The AUC for IL-Iβ was 0.80, and the cut-off value was > 47.2 pg/ml, with higher sensitivity (87.32 %) but lower specificity (61.54 %) than ESR and CRP. The results of the present study suggest that IL-1β may have an essential role in the diagnosis of IBD. ROC analysis showed significant discriminative abilities of RDW and NLR to differentiate between patients with IBD and healthy controls (AUC = 0.702 and AUC = 0.733) and cut-off values ≤ 34.6 and > 2.11, with a sensitivity of 52.24% and 67.16%, respectively. In their study, Okba et al. (2019) determined that the most effective NLR cut-off value for active UC was higher than 1.91, achieving a sensitivity of 90% and a specificity of 90%. Other selected parameters were also valuable for differentiating IBD from apparently healthy subjects. However, their AUC had lower sensitivities and specificities than the above parameters [27].

## Conclusions

We demonstrated for the first time in our region the validity of a panel of parameters as potential biomarkers for IBD. Findings concluded that patients with IBD, regardless of their type, have higher mean levels of ESR, CRP, FC, IL-6, IL-17A, IL-8, IL-1β, neutrophil number and %, NLR, and platelet number than apparently healthy subjects. In contrast, lymphocyte%, lymphocyte-mid-size leukocyte ratio (LMR), hemoglobin, MCH, hematocrit, and RDW are reduced in patients with IBD. FC is the highest-validity biomarker for IBD diagnosis, followed by IL-8 and ESR; therefore, both IL-8 and ESR can be used as additional essential markers. Despite the feasibility of the current findings, further studies should elaborate on the current findings. Assessment of genetic abnormalities in patients with IBD is mandatory to establish early diagnosis and effective treatment in their siblings. Further studies are required to investigate the impact of environmental factors on the development of IBD in this region and to evaluate the influence of current medical treatments on the levels of inflammatory biomarkers and the prognosis of IBD.
